# Distinct Types of Cell Death and the Implication in Diabetic Cardiomyopathy

**DOI:** 10.3389/fphar.2020.00042

**Published:** 2020-02-07

**Authors:** Yun Chen, Yuyun Hua, Xinshuai Li, Ishfaq Muhammad Arslan, Wei Zhang, Guoliang Meng

**Affiliations:** ^1^Department of Pharmacology, School of Pharmacy, Nantong University, Key Laboratory of Inflammation and Molecular Drug Target of Jiangsu Province, Nantong, China; ^2^School of Medicine, Nantong University, Nantong, China

**Keywords:** diabetic cardiomyopathy, cell death, apoptosis, autophagy, necrosis, entosis

## Abstract

Diabetic cardiomyopathy (DCM) is a chronic complication of diabetes mellitus, characterized by abnormalities of myocardial structure and function. Researches on the models of type 1 and type 2 diabetes mellitus as well as the application of genetic engineering technology help in understanding the molecular mechanism of DCM. DCM has multiple hallmarks, including hyperglycemia, insulin resistance, increased free radical production, lipid peroxidation, mitochondrial dysfunction, endothelial dysfunction, and cell death. Essentially, cell death is considered to be the terminal pathway of cardiomyocytes during DCM. Morphologically, cell death can be classified into four different forms: apoptosis, autophagy, necrosis, and entosis. Apoptosis, as type I cell death, is the fastest form of cell death and mainly occurs depending on the caspase proteolytic cascade. Autophagy, as type II cell death, is a degradation process to remove damaged proteins, dysfunctional organelles and commences by the formation of autophagosome. Necrosis is type III cell death, which contains a great diversity of cell death processes, such as necroptosis and pyroptosis. Entosis is type IV cell death, displaying “cell-in-cell” cytological features and requires the engulfing cells to execute. There are also some other types of cell death such as ferroptosis, parthanatos, netotic cell death, lysosomal dependent cell death, alkaliptosis or oxeiptosis, which are possibly involved in DCM. Drugs or compounds targeting the signals involved in cell death have been used in clinics or experiments to treat DCM. This review briefly summarizes the mechanisms and implications of cell death in DCM, which is beneficial to improve the understanding of cell death in DCM and may propose novel and ideal strategies in future.

## Introduction

Diabetic cardiomyopathy (DCM) is manifested as specific abnormalities of myocardial structure and function in diabetic patients without hypertension or coronary artery disease in clinic. DCM is regarded as the one of the most common complications of diabetes to increase the risk of heart failure (Paolillo et al., 2019). Hyperglycemia, insulin resistance, fatty acids, oxidative stress, inflammation, myocardial fibrosis and hypertrophy, mitochondrial dysfunction, endoplasmic reticulum stress, and endothelial dysfunction are possible molecular foundations for DCM (Wang et al., 2014; Cao et al., 2015; Chen et al., 2018; Li et al., 2018a). Reactive oxygen species (ROS) produced by fatty-acid oxidation or nicotinamide adenine dinucleotide phosphate (NADPH) oxidase induce cell death or tissue damage (Parim et al., 2019). Essentially, cell death is considered to be the terminal pathway of cardiomyocytes during DCM. Experimental models of DCM show that metabolic dysfunction, cardiac structural, or functional abnormalities are similar to the pathology of human DCM. Streptozotocin (STZ) is the most common agent to induce type 1 diabetes mellitus (T1DM). Zucker diabetic fatty rat, leptin receptor deficiency mice (db/db), and leptin deficiency (ob/ob) mice are useful models to study type 2 DM (T2DM). Recently, echocardiography, computed tomography, magnetic resonance imaging, and single-photon emission computed tomography are used for the diagnosis of DCM (Adingupu et al., 2019; Parim et al., 2019; Tan et al., 2019). *In vitro*, DCM is often imitated in rat embryonic heart derived H9C2 cells, neonatal cardiomyocytes or cardiac fibroblasts with high glucose (HG) or advance glycation end product (AGE) stimulation (Sun et al., 2019a; Tang et al., 2019b; Wang et al., 2019a).

**Figure 1:**
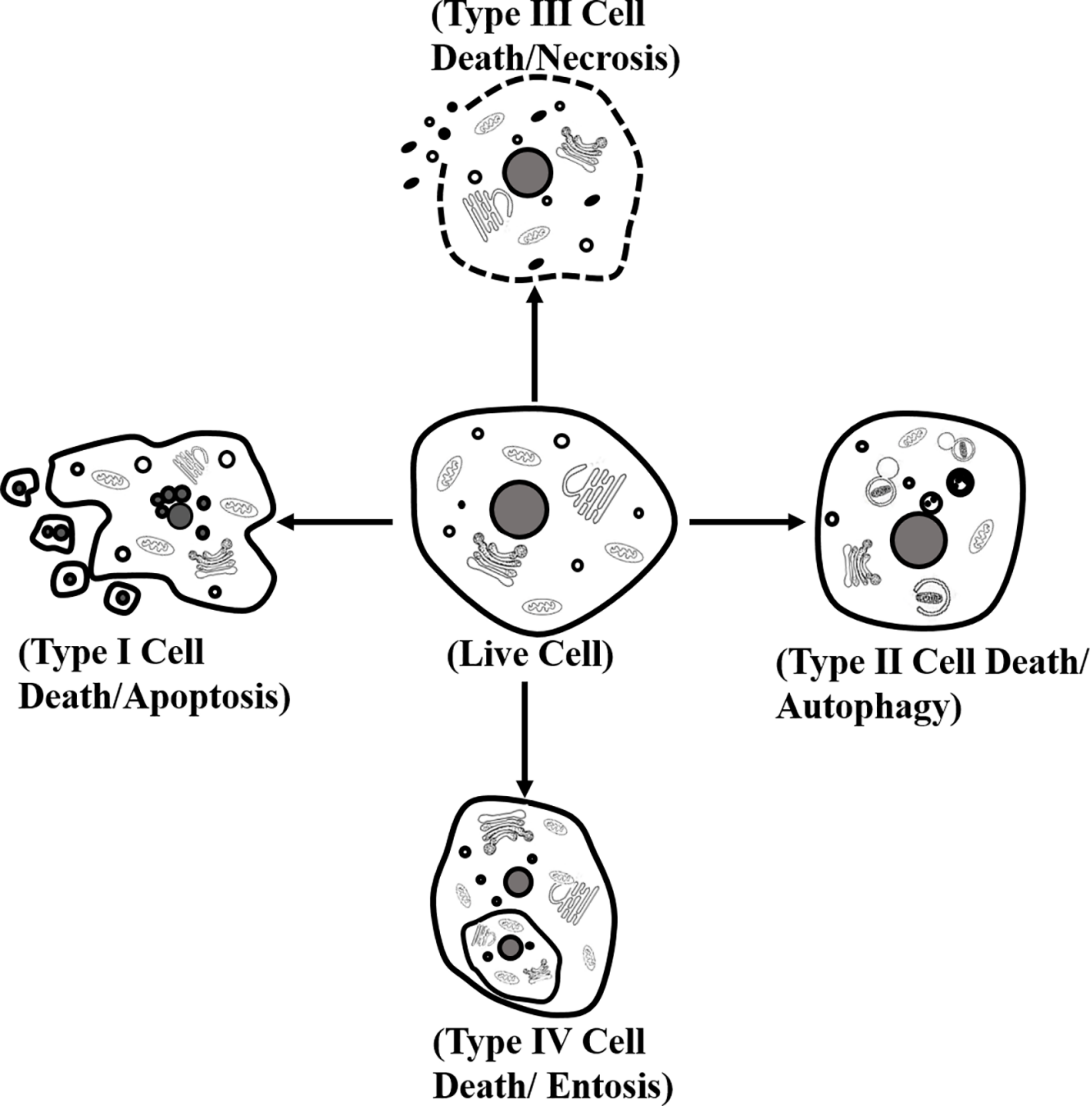
Classification of cell death. Morphologically, cell death is classified into four different forms: type I or apoptosis, type II or autophagy, type III or necrosis, and type IV or entosis.

A large number of studies have shown several distinct types of cardiomyocyte death. Morphologically, cell death can be classified into four different forms. (1) Type I or apoptosis, featuring with cytoplasmic shrinkage, nuclear pyknosis, karyorrhexis, DNA fragmentation, and plasma membrane blebbing, and eventually apoptotic body formation. (2) Type II, or autophagy, showing extensive cytoplasmic vacuolization to form autophagosome, phagocytosis and subsequent lysosomal degradation. (3) Type III or necrosis, manifesting with distinctive morphology, is different from type I and type II cell death. Its morphological changes contain organelle and plasma membrane rupture, finalizing with disposal of cell corpses absent of evident phagocytic and lysosomal involvement. (4) Type IV or entosis, displaying “cell-in-cell” cytological features in which cell winners engulf and kill cell losers (Martins et al., 2017; Tang et al., 2019a) (**Figure 1**). Biopsy showed that the apoptosis of a diabetic heart was 85 times higher than that of the nondiabetic heart, indicating that cardiomyocytes in diabetes were sensitive to suffer cell death (Cai and Kang, 2003). To understand the detailed mechanism of cell death in cardiomyocytes is beneficial to propose novel and ideal strategies for DCM.

## Mechanisms and Implications of Cell Death in DCM

### Apoptosis in DCM

**Figure 2:**
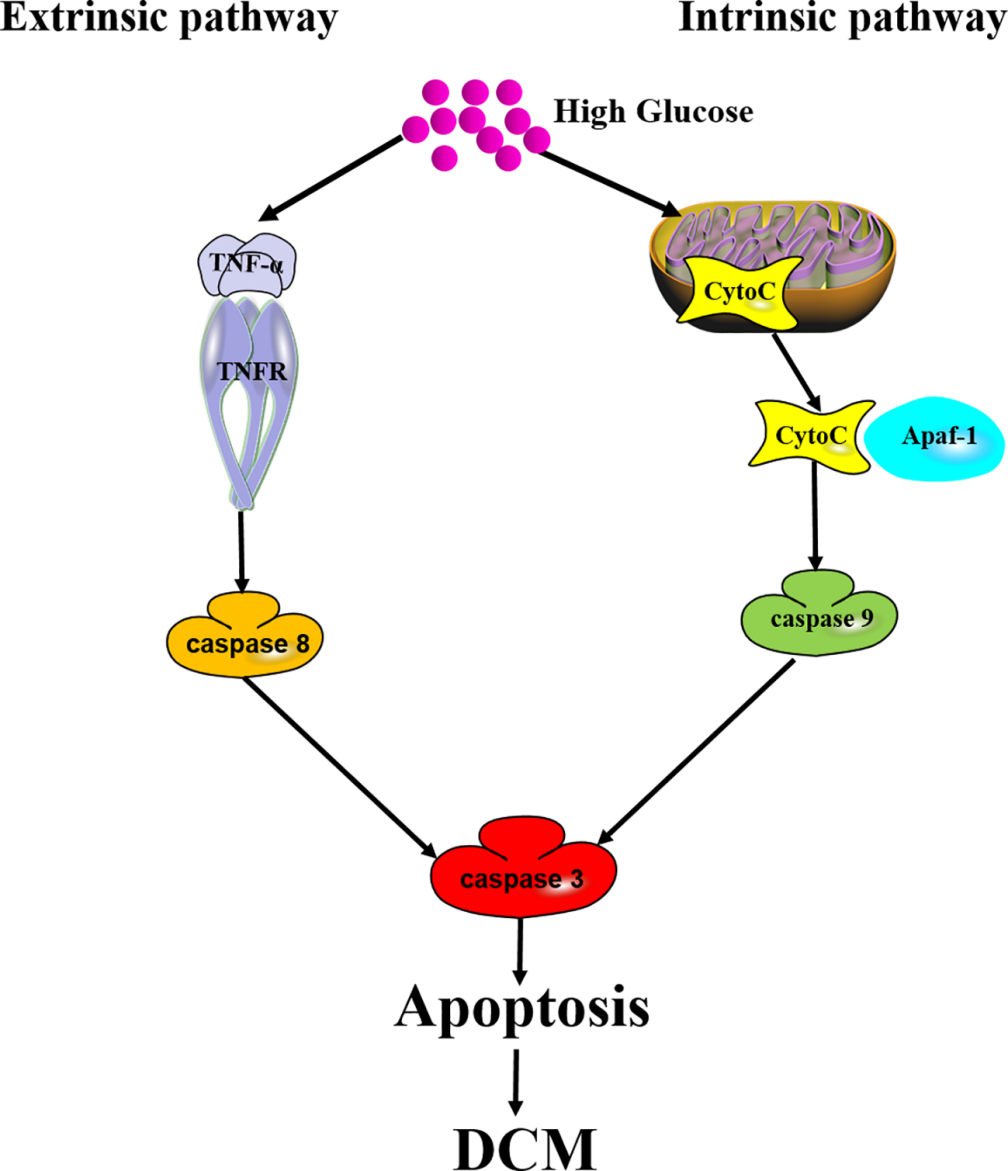
Mechanism of apoptosis in DCM. Both extrinsic apoptotic pathway and intrinsic apoptotic pathway are involved in DCM. Extrinsic pathway: long-term hyperglycemia triggers TNF-*α* to bind to TNF-*α* receptor (TNFR), initiates caspase 8 and subsequent caspase 3 activation, and finally results in apoptosis in the cardiomyocytes. Intrinsic pathway: Cyto C released into the cytosol combines with Apaf-1 to form a complex serving as a platform for caspase 9 and caspase 3 activation. Apoptosis induces cardiomyocyte cell loss to ultimately promote DCM.

Apoptosis is also called programmed cell death (PCD), which is the fastest form of cell death. Proteolytic cascade induced by caspases is the key biochemical characteristic of apoptosis. Caspases with an inactive proenzyme form are widely expressed in cardiomyocytes. Immediately after activation, a protease cascade is initiated by other procaspases depending on their proteolytic activity. This proteolytic cascade amplifies apoptotic pathways and finally results in rapid and irreversible cell death (Huang et al., 2019). There are two main pathways, including extrinsic apoptotic pathway and intrinsic apoptotic pathway, involved in the mechanisms of apoptosis. The extrinsic apoptotic pathway is also known as death receptor pathway, during which multiple death ligands, such as tumor necrosis factor-α (TNF-α) and Fas, bind to their homologous receptors to trigger cell death. Then, caspase 8 and caspase 9 are initiated to subsequently activate executioner caspases (caspase 3, caspase 6, and caspase 7), and finally result in apoptosis. The intrinsic pathway is known as mitochondrial pathway. During intrinsic pathway, a group of proteins called the complex permeability transition pore (CPTP) form a megapore and cover the inner and outer membranes of the mitochondria. Mitochondrial proteins such as cytochrome C (Cyto C), high temperature requirement protein A2 (HtrA2)/Omi, and second mitochondria-derived activator of caspases/direct inhibitors of apoptosis-binding protein with low pI (Smac/Diablo) are released into the cytosol. Then, Cyto C combines with apoptosis protease activating factor-1 (Apaf-1) to form a complex named “apoptosome”, which serves as a platform for the cleavage and activation of downstream caspases (D'Arcy, 2019). Activation of caspases ultimately leads to cell dismantling (**Figure 2**).

A great number of studies have demonstrated that long-term hyperglycemia in diabetic patients induces cardiomyocyte apoptosis (Joubert et al., 2019). Cardiomyocyte apoptosis leads to cell loss to decrease cardiac contractile function and to ultimately promote cardiac remodeling (Hu et al., 2017). It has been proven that astragalus polysaccharides (APS) protected HG induced-H9C2 cell apoptosis by reducing Cyto C release and inhibiting caspase activity. That is to say APS has the potential ability to attenuate DCM through suppressing both extrinsic and intrinsic apoptotic pathways (Sun et al., 2017). One study reported that nicorandil decreased TdT-mediated DUTP nick end labeling (TUNEL)-positive cells, B-cell lymphoma-2 (bcl-2)-associated X (bax) as well as cleaved caspase 3 expression, while it increased bcl-2 expression in the heart of diabetic rats. Moreover, 5-HD, the competitive antagonist of nicorandil, increased apoptosis but reduced the phosphorylation of phosphatidylinositol 3-kinase (P13K), protein kinase B (AKT), endothelial nitric oxide synthase (eNOS), and mammalian target of rapamycin (mTOR) in H9C2 cardiomyocyte with high glucose stimulation. These data demonstrated that nicorandil alleviated hyperglycemia-caused cardiomyocyte apoptosis (Wang et al., 2019c). Li et al. found that long noncoding RNAs H19/MicroRNA-675 axis was involved in HG-induced cardiomyocyte apoptosis by downregulation of voltage-dependent anion channel 1 (VDAC1), which was required for the mitochondria-mediated apoptosis (Li et al., 2016). Recent studies also demonstrated that miR-186-5p overexpression suppressed apoptosis in HG-treated cardiomyocytes (Liu et al., 2019). Chronic and severe endoplasmic reticulum (ER) stress also resulted in cell apoptosis. APS provided cardioprotective effects on DCM by inhibiting cardiomyocytes apoptosis *via* suppressing protein kinase RNA-like ER kinase (PERK) and activating transcription factor 6 (ATF6)-related pathway of ER stress (Sun et al., 2019c). Matrine induced caspase 3 and caspase 9 down-regulation along with bcl-2 and p53 up-regulation. In addition, matrine administration inhibited the transforming growth factor beta (TGF-β) induced PERK signal pathway activation, which was involved in ER stress-induced apoptosis (Hou et al., 2019). Together, these events suggest that apoptosis promotes cardiomyocyte damage during DCM through multiple upstream signal pathways. Thus, compounds or molecules inhibiting apoptosis may serve as potential therapeutic agents for DCM.

### Autophagy in DCM

Autophagy is initially described as a degradation process to eliminate damaged proteins and dysfunctional organelles. Autophagy is usually induced by nutrient deprivation, hypoxia, oxidative stress, genotoxic stress, or high glucose. Autophagy commences by autophagosome formation, a double-membrane vesicle swallowing up cytoplasmic materials (Meng et al., 2019). Autophagy is tightly regulated by autophagy-related (ATG) proteins, which is encoded by a family of highly conserved genes. Multiple signaling pathways including adenosine 5′-monophosphate (AMP)-activated protein kinase (AMPK), mTOR, unc-51-like kinase 1 (ULK1), PI3K/AKT, GTPases, calcium, and protein synthesis are involved in autophagy (Yang et al., 2005; Li et al., 2017; Bootman et al., 2018; Li et al., 2018b; Wang et al., 2019b).

The real role of autophagy on DCM is quite conflicting. Various studies have demonstrated the cardioprotective effect of autophagy in the heart of diabetic animals (Bhattacharya et al., 2018). In a study that explored the effect of aldehyde dehydrogenase 2 (ALDH2) on diabetes-induced myocardial dysfunction, there was decreased ratio of microtubule-associated protein 1 light chain 3-II (LC3II)-to-LC3I, reduced autophagy related 7 (ATG7) expression but increased sequestosome 1 (p62) levels, suggesting depressed autophagy in diabetes. ALDH2 overexpression or its agonist Alda-1 improved autophagy to reverse diabetes or high glucose-induced dysfunctions *via* AMPK and forkhead box O3a (FOXO3a) (Guo et al., 2015). Sirtuin3 (Sirt3) deficiency decreased LC3 puncta, autophagosomes, and LC3II level, *via* acetylated-Foxo3A enhancement and parkin inhibition, indicating that Sirt3 deficiency exacerbates diabetic cardiac dysfunction (Yu et al., 2017). Activating cannabinoid receptor 2 (CB2) produced a cardioprotective effect in DCM as well as cardiomyocytes under HG challenge through inducing AMPK-mTOR-p70S6K signaling-mediated autophagy (Wu et al., 2018a). Surprisingly, another study suggested that diminished autophagy gave adaptive response to limit diabetic cardiac injury in type 1 diabetic mice (Xu et al., 2013). Consistently, diabetes-associated myocardial mitochondrial dysfunction was associated with enhanced autophagy in the myocardium of diabetic Goto-Kakizaki rats (Liu et al., 2014). Hence, determining whether autophagy is adaptive or maladaptive is critical for the execution of efficient therapeutic treatment for DCM.

### Necrosis in DCM

Necrotic cell death contains a great diversity of cell death processes. Necrosis may occur in the case of extensive damage such as high temperature and mechanical stress, leading to cell integrity destruction (Marunouchi and Tanonaka, 2015). In this case, necrosis is passive and does not require any specific signaling pathways. Another type of necrosis, called secondary necrosis, occurs at the late stage of apoptosis or autophagy when dead cells can't be removed by phagocytosis. Secondary necrosis is regarded as independent of initial signal events such as apoptosis or autophagy (Wu et al., 2018b). However, necrosis can also be a result of signaling cascade. Early study showed that diabetes increased necrosis by fourfold in cardiomyocytes (Frustaci et al., 2000).

**Figure 3:**
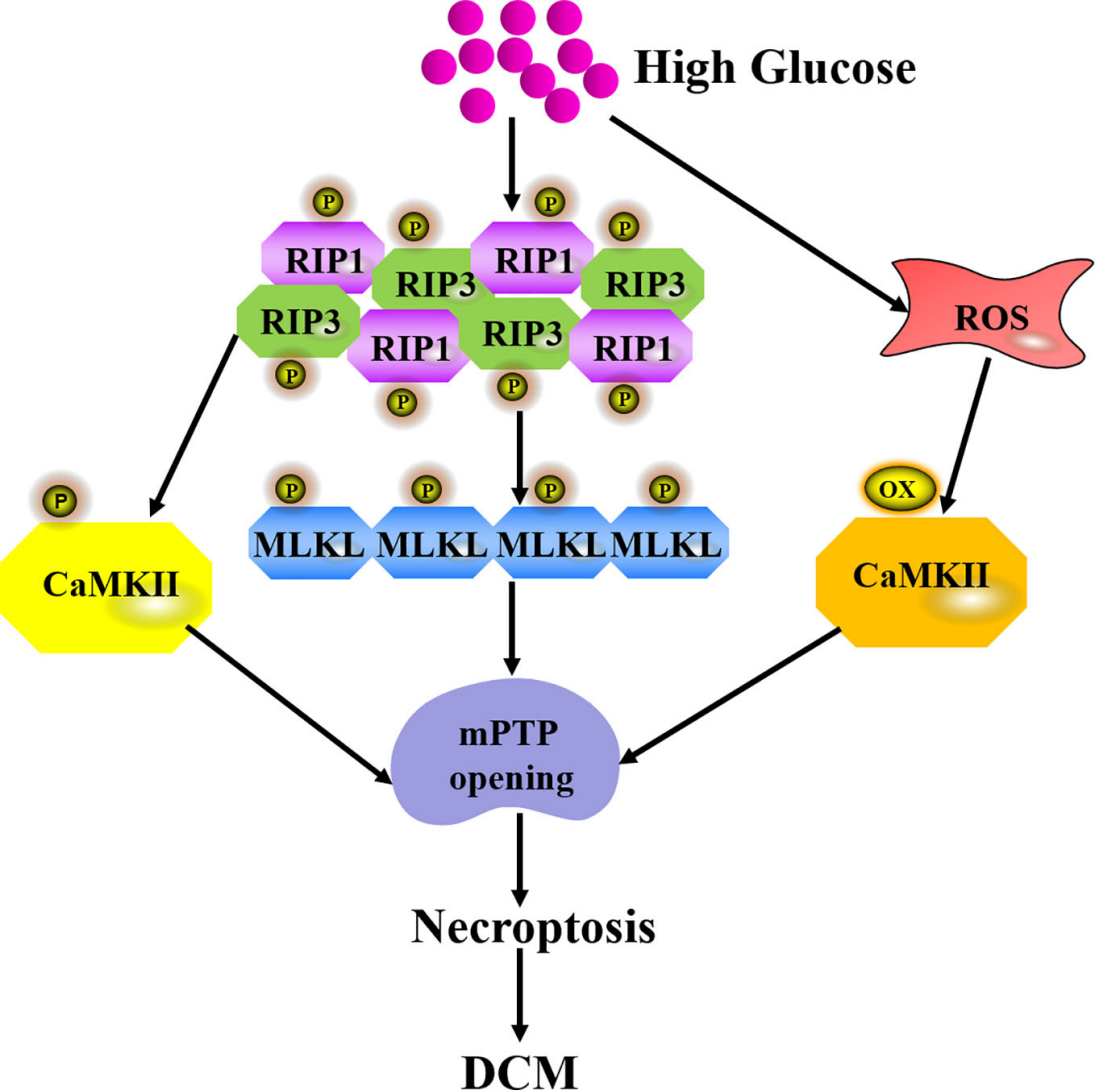
Mechanism of necroptosis in DCM. Once recruited by RIP1 after HG exposure, RIP3 is activated by auto-phosphorylation to promote the recruitment and activation of mixed lineage kinase domain like protein (MLKL). RIP3 also phosphorylates CaMKII to induce mPTP opening. High glucose also increases ROS to activate CaMKII by oxidation and finally triggers mPTP opening, which is a final pathway of necroptosis during DCM.

Necroptosis is the best-characterized form of programmed necrosis, showing features of both apoptosis and necrosis (Tang et al., 2019a). The pathway of necroptosis activation after high glucose stimulation is mediated by ligand to death receptors such as tumor necrosis factor receptor 1 (TNFR1) and Fas receptors. Damage-associated molecular patterns (DAMPs), nucleotide-binding, and oligomerization domain (NOD)-like receptors (NLRs) ripoptosome, and protein kinase R (PKR) complexes also trigger necroptosis. TNFR1-mediated necroptosis is the most thoroughly studied pathway to activate necroptosis. After TNF-α binds to TNFR1, there are three possible divergent functions including cell survival, apoptosis, or necroptosis *via* different signaling complexes. Complex I is prosurvival and Complex IIa is proapoptotic. Complex IIb formation results in necroptosis (Galluzzi et al., 2018). Receptor-interacting protein 3 (RIP3) is regarded as a critical regulator of necroptosis. RIP3 regulates necroptosis on a RIP1-dependent manner in diabetes. Once recruited by RIP1 after HG exposure, RIP3 is activated by auto-phosphorylation to promote mixed lineage kinase domain like protein (MLKL) phosphorylation. Then, MLKL oligomerizes and translocates to the cell membrane, interacts with phosphatidylinositol lipids and cadiolipin to result in membrane permeabilization (Gupta et al., 2018). Calmodulin-dependent protein kinase II (CaMKII) is a newly found RIP3 substrate to induce necroptosis. CaMKII is abundant in the myocardium and is inactivated under normal conditions. CaMKII phosphorylation by intracellular Ca^2+^ or RIP1 facilitates necroptosis (Nomura et al., 2014). Meanwhile, HG also increases ROS to activate CaMKII by oxidation (Feng and Anderson, 2017). Altogether, the above pathological changes trigger the opening of mitochondrial permeability transition pore (mPTP), which is involved in the final pathway of necroptosis (**Figure 3**). Some research found that knockdown of cyclophilin D (CypD), a protein that increases mPTP opening probability, protected against RIP3-induced cardiomyocyte necrosis (Zhang et al., 2016). RIP3 up-regulation increased phosphoglycerate mutase 5 (PGAM5) expression, enhanced CypD phosphorylation, and finally resulted in mPTP opening. Excessive opening of the mPTP also amplified the death signal and ultimately led to necroptosis in the endothelial cells (Zhou et al., 2018a).

It was observed that when H9C2 cells were exposed to high glucose, cell viability, the activity of ALDH2 mRNA or protein expression were suppressed, while ROS levels as well as RIP1, RIP3, and MLKL expression were enhanced. Necrostain-1 (Nec-1, a specific inhibitor of necroptosis) or Alda-1 (the ALDH2 activator) attenuated HG-induced down-regulation of ALDH2 and enhancement of RIP1, RIP3 and MLKL. In general, ALDH2 activation protected against HG-induced H9C2 cell injury, partly by inhibiting necroptosis (Fang et al., 2018). Another study showed that HG markedly increased the expression of Toll-like receptor 4 (TLR4), which was attenuated by NAC, a ROS scavenger. HG also significantly increased the expression of RIP3, which was ameliorated by TAK-242 (an inhibitor of TLR4) or Nec-1. In addition, NAC mitigated HG-induced RIP3 expression up-regulation. More importantly, DZ (a mitochondrial K_ATP_ channel opener) and Pin (a non-selective K_ATP_ channel opener) reduced the increased levels of TLR4 and RIP3 induced by HG. Taken together, the opening of K_ATP_ channels inhibited necroptosis through ROS-TLR4 pathway to protect H9C2 cells against HG-induced damage (Liang et al., 2017). Our recent research revealed that inhibitor 1 of protein phosphatase 1 (I1PP1) over-expression alleviated CaMKIIδ alternative splicing disorder, suppressed ROS overproduction, inhibited CaMKII oxidation, suppressed necroptosis, and ultimately alleviated high glucose-induced cardiomyocyte injury (Sun et al., 2019a).

Pyroptosis is another novel form of programmed necrosis (Robinson et al., 2019). It is responsible for cellular lysis and extracellular release of proinflammatory cytokines such as interleukin-1*β* (IL-1β) and interleukin-18 (IL-18) by HG stimulation (Zeng et al., 2019). There are two different pathways including the canonical pathway and noncanonical pathway in pyroptosis. In the canonical pathway, cytoplasmic multiprotein complexes, named inflammasome, consist of the nucleotide-binding oligomerization domain (NOD)-like receptor (NLR) family (including NLRP3, NLRP1, NLRC4, NLRP9 and NLRP6), the pyrin and HIN domain (PYHIN) protein families (absent in melanoma 2, AIM2), and pyrin proteins. Distinct inflammasomes are recognized by pathogen-associated molecular patterns (PAMPs) or DAMPs to activate caspase 1, leading to pyroptosis (Lee et al., 2019). In the noncanonical pathway, bacterial lipopolysaccharide (LPS) is delivered to the cytosol to activate caspase 11. Activated caspase 11 directly induces pyroptosis. The cleaved caspase 11 also activates the Gasdermin-D (GSDMD) to form a membrane pore. Meanwhile, the NLRP3 inflammasome is activated by the N-terminal fragment of GSDMD (GSDMD-N) to trigger pyroptosis *via* the canonical pathway (Zhao et al., 2019).

**Figure 4:**
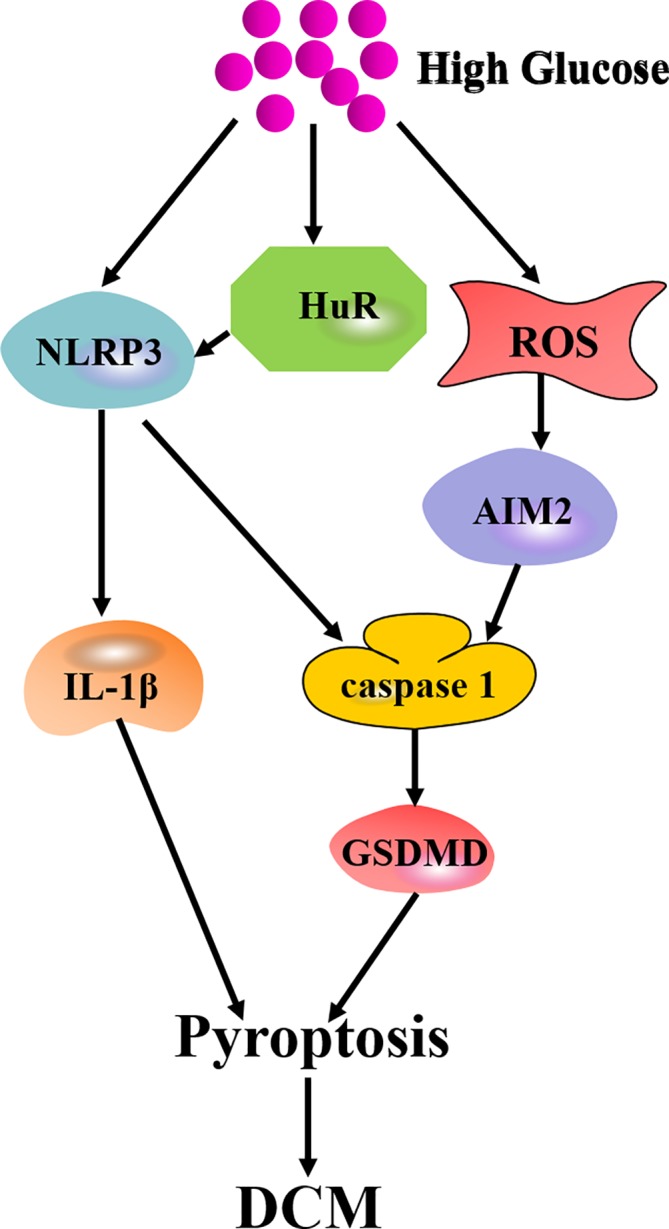
Mechanism of pyroptosis in DCM. High glucose augments expression of NLRP3 and HuR to activate IL-1*β* or caspase 1/GSDMD-mediated pyroptosis. High glucose also elevates ROS production to increase AIM2 expression and ultimately mediates pyroptosis through caspase 1/GSDMD pathway.

Emerging evidence has verified that pyroptosis is involved in the progression of DCM. Jeyabal et al. found that NLRP3, caspase 1, and ELAV-like RNA binding protein 1 (ELAVL1, also called Hu-Antigen, HuR) expression in both human diabetic heart and HG-exposed human ventricular cardiomyocytes were augmented. Further study identified HuR as a direct target of miR-9. MiR-9 mimic transfection reduced HuR, caspase 1, and IL-1β expression to inhibit HG-induced pyroptosis in human cardiomyocytes. This study highlighted that targeting miR-9/HuR would play a therapeutic role in cell death during DCM (Jeyabal et al., 2016). Recently, Wang et al. found that absent in melanoma 2 (AIM2) inflammasome, caspase-1, IL-1β, and GSDMD were elevated in the myocardium of diabetes mellitus. ROS was also increased in H9C2 cells with HG stimulation. AIM2-shRNA reduced caspase 1 and IL-1β expression, suppressed GSDMD-N level and attenuated HG-induced pyroptosis. Furthermore, AIM2 level dropped significantly if ROS was inhibited. This study indicated that AIM2 inhibition is beneficial to attenuate diabetic cardiomyopathy *via* alleviating GSDMD-N-related pyroptosis (Wang et al., 2019d) (**Figure 4**). These findings confirmed the distinctive role of pyroptosis in DCM. To inhibit the pyroptosis signaling pathways is to capably expand the potential therapeutic targets for DCM treatment.

### Other Types of Cell Death in DCM

Entosis seems fundamentally different from most typical forms of cell death due to the requirement of engulfing cells to execute. Entosis is mediated by cellular engulfment *via* E-cadherin, *α*-catenin, Rho family of GTPases, and rho-associated kinase (ROCK). Entosis is identified as an essential mechanism of embryo implantation (Li et al., 2015), which plays pro- and antitumorigenic roles in cancer. However, no data regarding the role of entosis in cardiovascular diseases has been found until now, and the possible mechanisms were incompletely defined.

Ferroptosis is an iron- and lipotoxicity-dependent form of regulated cell death (RCD). It is characterized by small mitochondria with reduced crista and condensed or ruptured outer membrane (Baba et al., 2018). Acyl-CoA synthetase long-chain family member 4 (ACSL4), lysophosphatidylcholine acyltransferase 3 (LPCAT3), and arachidonate lipoxygenase (ALOXs) pathways mediate the oxidation of polyunsaturated fatty acids, which is necessary for the lipid toxicity of ferroptosis (Dixon et al., 2015; Chu et al., 2019; Xiao et al., 2019). ACSL4 up-regulation is the marker of ferroptosis (Doll et al., 2017). Decreasing antioxidant glutathione or inhibiting glutathione peroxidases inevitably enhanced ROS formation by erastin, a cell-permeable ferroptosis activator (Yang et al., 2014). Ferroptosis has been reported to be regulated during drug therapy; whether it participates in the pathology of DCM is unclear. In consideration of ROS formation promoting ferroptosis, there is a serious possibility that ferroptosis is involved in DCM.

Parthanatos is a poly ADP-ribose (PAR) polymerase-1 (PARP-1)-dependent type of cell death, which is activated by oxidative stress-induced DNA damage (Wang et al., 2019e). Neither apoptotic body formation nor DNA fragmentation is observed during parthanatos. Parthanatos does not produce cell swelling or lysosomal degradation. Energy exhaustion and apoptosis-inducing factor (AIF) release from the mitochondria mediated by PAR or calpain are possibly the main mechanisms of parthanatos. Parthanatos has been reported to be involved in cardiovascular diseases, renal diseases, diabetes, and neurodegeneration (Linkermann, 2016; Barany et al., 2017; Kam et al., 2018; Aizawa et al., 2019; Li et al., 2019d). PARP inhibitor L-2286 prevented cardiac remodeling, improved systolic function, and delayed the development of heart failure (Bartha et al., 2009). Thus, PARP inhibition may have the potential ability to attenuate DCM.

Netotic cell death is a form of RCD driven by neutrophil extracellular traps (NETs), which is regulated by NADPH oxidase-mediated ROS production and histone citrullination. NET formation and release, or NETosis relies on ROS production, autophagy, granular enzyme release, and translocation from the cytosol to the nucleus. Histone citrullination eventually leads to chromatin depletion, nuclear membrane destruction, and chromatin fiber release (Hemmers et al., 2011; Mitroulis et al., 2011; Tang et al., 2019a). Efforts could be endeavored to explore the mechanisms of NETosis underlying DCM, thereby providing new therapeutic policies for DCM.

Lysosomal-dependent cell death (LCD), also known as lysosomal cell death, is a form of regulated cell death mediated by intralysosomal components or iron translocation resulting from lysosomal membrane permeabilization (LMP) to amplify or initiate cell death during apoptosis, autophagy, and ferroptosis (Wang et al., 2018). Although the relation between LCD and DCM was easy to be deduced, further studies are still indispensable.

Alkaliptosis is driven by intracellular alkalization. It is known that down-regulation of inhibitor of nuclear factor kappa B kinase subunit beta (IKBKB) and nuclear factor-*κ*B (NF-*κ*B) induces alkaliptosis (Song et al., 2018). The pathological significance of alkaliptosis in human diseases remains complicated, and the main signals of allkaliptosis need to be illuminated in the future.

Oxeiptosis is a novel caspase-independent RCD induced by ROS, which is mediated through Kelch-like ECH-associated protein 1 (KEAP1)/PGAM5/apoptosis-inducing factor 1 mitochondrial (AIFM1) pathway (Scaturro and Pichlmair, 2018). Since ROS accumulation links to multiple physiological and pathological processes, it is very likely that oxeiptosis promotes the development of several diseases including DCM.

Different types of cell death are mediated by distinct but overlapping central pathways. One study found that autophagy was inhibited but apoptosis was amplified in diabetic heart (Xing et al., 2019). Elevated bax/bcl-2 ratio, increased cleaved caspase 3 level, augmented NLRP3, cleaved caspase 1, IL-1β, and cleaved GSDMD expression along with increased ROS production were detected in the myocardium of db/db mice, indicating the involvement of apoptosis and pyroptosis in DCM (Xue et al., 2019). Altogether, several types of cell death may coexist in the occurrence and development of DCM. Moreover, apoptosis, necrosis, necroptosis, pyroptosis, autophagy, or ferroptosis might act simultaneously during DCM. Inhibition of one kind of cell death may possibly promote other types of cell death and cause compensation, which eventually increases the complexity of pathogenesis about DCM and enhance difficulty of treatment for DCM.

## Conclusion and Prospective

Current therapeutic strategies for DCM involve insulin and insulin-secreting agents, oral antihyperglycemic medication, *β*-blockers, angiotensin converting enzyme inhibitors, angiotensin II receptor antagonists, calciumion channel antagonists, and hydroxymethylglutaryl CoA reductase inhibitors. However, the above synthetic drugs have many adverse effects including gastrointestinal disturbances, hepatotoxicity, abdominal pain, genitourinary tract infection, and so on (Parim et al., 2019). As cell death is a determinant pathological fate in DCM, the executors of cell death signal pathways are obviously potential therapeutic targets for DCM. Many drugs or compounds are being used in or under clinical trials. Take some examples: Metformin, a common antidiabetic drug, activates AMPK and improves autophagy *via* inhibiting the mTOR pathway and alleviating pyroptosis (Yang et al., 2019). Crocin, a carotenoid extracted from saffron, improves the deteriorated cardiac function in diabetic animals by inhibiting apoptosis and normalizing autophagy (Feidantsis et al., 2018). Protein phosphatase 2A (PP2A) is a central cardiac phosphatase that regulates the functions of diverse myocytes through target molecules. Okadaic acid (OA), an inhibitor of PP2A, inhibited apoptosis of experimental diabetic mellitus-related cardiomyopathy (Guan et al., 2019). These researches will improve the understanding of cell death in DCM and set the stage for novel therapies in future. Several drug or compounds targeting the signals in cell death have been used in clinics or experiments for DCM (**Table 1**).

**Table 1:** Drugs or compounds against cell death in diabetic cardiomyopathy.

Drugs or compounds	Targets of cell death type	Effects	References
Astragalus Polysaccharides	apoptosis	oxidative stress↓, antioxidant capacity↑	(Sun et al., 2019b)
Catalpol	apoptosis	bax/bcl-2↓, cleaved caspase 3↓	(Zou et al., 2019b)
Crocim	autophagy	LC3II/LCI↑	(Feidantsis et al., 2018)
	apoptosis	bax/bcl-2↓, cleaved caspase 3↓	
Curcumin	apoptosis	cleaved caspase 3↓, cytochrome C↓, bcl-2↑	(Yao et al., 2018)
	autophagy	LC3II/LC3I↑, p62↓	
Dexmedetomidine	autophagy	ATG7↓	(Oh et al., 2019)
Dihydromyricetin	autophagy	LC3II/LC3I↑, p62↓, ATG7↓	(Wu et al., 2017)
	apoptosis	bax/bcl-2↓, cleaved caspase3↓, cleaved caspase 9↓	
1,25-Dihydroxyvitamn-D3	autophagy	LC3II/LC3I↑	(Wei et al., 2017)
Empagliflozin	apoptosis	bax/bcl-2↓, cleaved caspase 3↓	(Xue et al., 2019)
	pyroptosis	NLRP3↓, caspase 1↓, IL-1β↓, cleaved GSDMD↓, full-length GSDMD↑	
GYY413	apoptosis	bax/bcl-2↓, caspase 3↓	(Li et al., 2019b)
Helix B surface peptide	autophagy	LC3II/LC3I↑, p62↓	(Lin et al., 2017)
Metformin	pyroptosis	NLRP3↓, caspase 1↓, IL-1β↓, GSDMD-N↓	(Zou et al., 2019a)
NaHS	necroptosis	RIP3↓, cleaved caspase 3↓	(Liang et al., 2016)
Nicorandil	apoptosis	bax/bcl-2↓, cleaved caspase 3↓	(Wang et al., 2019c)
Notoginsenoside R1	apoptosis	mitochondrial membrane depolarization↓, ROS↓	(Zhang et al., 2018)
Okadaic acid	apoptosis	bax/bcl-2↓, cleaved caspase 3↓	(Ren et al., 2016)
Palbociclib	apoptosis	bax/bcl-2↓, caspase 3↓	(Wang et al., 2019f)
Piceatonnol	apoptosis	bax/bcl-2↓, caspase 3↓	(Li et al., 2019a)
Resveratrol	autophagy	LC3II/LC3I↑, p62↓	(Xu et al., 2018)
	apoptosis	cleaved caspase 3↓	
Sitagliptin	autophagy	LC3II/LC3I↑, p62↓	(Zhou et al., 2018b)
Sophocarpine	apoptosis	bax/bcl-2↓, cleaved caspse3↓, cleaved caspase 9↓	(Zou et al., 2019a)
Vaspin	autophagy	LC3II/LC3I↑, p62↓	(Li et al., 2019c)

Taken together, cell death is the terminal pathway of cardiomyocytes during DCM. Although upstream signal regulation seems to attenuate cell death effectively, it may produce a series of nonspecific effects. Targeting cell death directly may be more practicable than its upstream signaling pathway.

## Author Contributions

YC researched documents and wrote the manuscript. YH, XL, IA, and WZ revised the manuscript. GM designed the study and reviewed the manuscript. All authors approved the final version of the manuscript.

## Funding

The work was funded by grants (81770279, 81670243 and 81873470) from the National Natural Science Foundation of China, a major project of Natural Science Research in Jiangsu Higher Education Institutions (18KJA310005), the Six Talent Peaks Project in Jiangsu Province (2018-WSN-062), grants from the China Postdoctoral Science Foundation (2017M610342 and 2019T120449), a Jiangsu Planned Project for Postdoctoral Research Funds (1701050A), a Research and Innovation Project of Graduate Students in Jiangsu Province (KYCX18_2401), a Science and Technology Project of Taicang City (TC2019KJFZ02), and the Nantong University Cooperative Innovation Program of Small Molecular Compound R&D (NTU2016-1).

## Conflict of Interest

The authors declare that the research was conducted in the absence of any commercial or financial relationships that could be construed as a potential conflict of interest.

## References

[B1] Adingupu D. D. Gopel S. O. Gronros J. Behrendt M. Sotak M. Miliotis T. (2019). SGLT2 inhibition with empagliflozin improves coronary microvascular function and cardiac contractility in prediabetic ob/ob(-/-) mice. Cardiovasc. Diabetol. 18, 16. 10.1186/s12933-019-0820-6 30732594 PMC6366096

[B2] Aizawa S. Brar G. Tsukamoto H. (2019). Cell death and liver disease. Gut. Liver. 14, 20–29. 10.5009/gnl18486 PMC6974333 30917630

[B3] Baba Y. Higa J. K. Shimada B. K. Horiuchi K. M. Suhara T. Kobayashi M. (2018). Protective effects of the mechanistic target of rapamycin against excess iron and ferroptosis in cardiomyocytes. Am. J. Physiol. Heart Circ. Physiol. 314, H659–H668. 10.1152/ajpheart.00452.2017 29127238 PMC5899260

[B4] Barany T. Simon A. Szabo G. Benko R. Mezei Z. Molnar L. (2017). Oxidative stress-related parthanatos of circulating mononuclear leukocytes in heart failure. Oxid. Med. Cell. Longev. 2017, 1249614. 10.1155/2017/1249614 29250299 PMC5700485

[B5] Bartha E. Solti I. Kereskai L. Lantos J. Plozer E. Magyar K. (2009). PARP inhibition delays transition of hypertensive cardiopathy to heart failure in spontaneously hypertensive rats. Cardiovasc. Res. 83, 501–510. 10.1093/cvr/cvp144 19443425

[B6] Bhattacharya D. Mukhopadhyay M. Bhattacharyya M. Karmakar P. (2018). Is autophagy associated with diabetes mellitus and its complications? A review. EXCLI. J. 17, 709–720. 10.17179/excli2018-1353 30190661 PMC6123605

[B7] Bootman M. D. Chehab T. Bultynck G. Parys J. B. Rietdorf K. (2018). The regulation of autophagy by calcium signals: Do we have a consensus? Cell Calcium. 70, 32–46. 10.1016/j.ceca.2017.08.005 28847414

[B8] Cai L. Kang Y. J. (2003). Cell death and diabetic cardiomyopathy. Cardiovasc. Toxicol. 3, 219–228. 10.1385/CT:3:3:219 14555788

[B9] Cao H. Chen T. Shi Y. (2015). Glycation of human serum albumin in diabetes: impacts on the structure and function. Curr. Med. Chem. 22, 4–13. 10.2174/0929867321666140912155738 25245514

[B10] Chen X. F. Li X. L. Yang M. Song Y. Zhang Y. (2018). Osteoprotective effects of salidroside in ovariectomized mice and diabetic mice. Eur. J. Pharmacol. 819, 281–288. 10.1016/j.ejphar.2017.12.025 29242120

[B11] Chu B. Kon N. Chen D. Li T. Liu T. Jiang L. (2019). ALOX12 is required for p53-mediated tumour suppression through a distinct ferroptosis pathway. Nat. Cell Biol. 21, 579–591. 10.1038/s41556-019-0305-6 30962574 PMC6624840

[B12] D'Arcy M. S. (2019). Cell death: a review of the major forms of apoptosis, necrosis and autophagy. Cell Biol. Int. 43, 582–592. 10.1002/cbin.11137 30958602

[B13] Dixon S. J. Winter G. E. Musavi L. S. Lee E. D. Snijder B. Rebsamen M. (2015). Human haploid cell genetics reveals roles for lipid metabolism genes in nonapoptotic cell death. ACS Chem. Biol. 10, 1604–1609. 10.1021/acschembio.5b00245 25965523 PMC4509420

[B14] Doll S. Proneth B. Tyurina Y. Y. Panzilius E. Kobayashi S. Ingold I. (2017). ACSL4 dictates ferroptosis sensitivity by shaping cellular lipid composition. Nat. Chem. Biol. 13, 91–98. 10.1038/nchembio.2239 27842070 PMC5610546

[B15] Fang T. Cao R. Wang W. Ye H. Shen L. Li Z. (2018). Alterations in necroptosis during ALDH2mediated protection against high glucoseinduced H9c2 cardiac cell injury. Mol. Med. Rep. 18, 2807–2815. 10.3892/mmr.2018.9269 30015964 PMC6102746

[B16] Feidantsis K. Mellidis K. Galatou E. Sinakos Z. Lazou A. (2018). Treatment with crocin improves cardiac dysfunction by normalizing autophagy and inhibiting apoptosis in STZ-induced diabetic cardiomyopathy. Nutr. Metab. Cardiovasc. Dis. 28, 952–961. 10.1016/j.numecd.2018.06.005 30017436

[B17] Feng N. Anderson M. E. (2017). CaMKII is a nodal signal for multiple programmed cell death pathways in heart. J. Mol. Cell. Cardiol. 103, 102–109. 10.1016/j.yjmcc.2016.12.007 28025046 PMC5404235

[B18] Frustaci A. Kajstura J. Chimenti C. Jakoniuk I. Leri A. Maseri A. (2000). Myocardial cell death in human diabetes. Circ. Res. 87, 1123–1132. 10.1161/01.RES.87.12.1123 11110769

[B19] Galluzzi L. Vitale I. Aaronson S. A. Abrams J. M. Adam D. Agostinis P. (2018). Molecular mechanisms of cell death: recommendations of the nomenclature committee on cell death 2018. Cell Death Differ. 25, 486–541. 10.1038/s41418-017-0012-4 29362479 PMC5864239

[B20] Guan Y. Zhou L. Zhang Y. Tian H. Li A. Han X. (2019). Effects of PP2A/Nrf2 on experimental diabetes mellitus-related cardiomyopathy by regulation of autophagy and apoptosis through ROS dependent pathway. Cell. Signal. 62, 109339. 10.1016/j.cellsig.2019.06.004 31173878

[B21] Guo Y. Yu W. Sun D. Wang J. Li C. Zhang R. (2015). A novel protective mechanism for mitochondrial aldehyde dehydrogenase (ALDH2) in type i diabetes-induced cardiac dysfunction: role of AMPK-regulated autophagy. Biochim. Biophys. Acta 1852, 319–331. 10.1016/j.bbadis.2014.05.017 24874076

[B22] Gupta K. Phan N. Wang Q. Liu B. (2018). Necroptosis in cardiovascular disease - a new therapeutic target. J. Mol. Cell. Cardiol. 118, 26–35. 10.1016/j.yjmcc.2018.03.003 29524460 PMC5940532

[B23] Hemmers S. Teijaro J. R. Arandjelovic S. Mowen K. A. (2011). PAD4-mediated neutrophil extracellular trap formation is not required for immunity against influenza infection. PloS One 6, e22043. 10.1371/journal.pone.0022043 21779371 PMC3133614

[B24] Hou H. Zhang Q. Dong H. Ge Z. (2019). Matrine improves diabetic cardiomyopathy through TGF-beta-induced protein kinase RNA-like endoplasmic reticulum kinase signaling pathway. J. Cell. Biochem. 120, 13573–13582. 10.1002/jcb.28632 30938856

[B25] Hu X. Bai T. Xu Z. Liu Q. Zheng Y. Cai L. (2017). Pathophysiological fundamentals of diabetic cardiomyopathy. Compr. Physiol. 7, 693–711. 10.1002/cphy.c160021 28333387

[B26] Huang M. L. Chiang S. Kalinowski D. S. Bae D. H. Sahni S. Richardson D. R. (2019). The role of the antioxidant response in mitochondrial dysfunction in degenerative diseases: cross-talk between antioxidant defense, autophagy, and apoptosis. Oxid. Med. Cell. Longev. 2019, 6392763. 10.1155/2019/6392763 31057691 PMC6476015

[B27] Jeyabal P. Thandavarayan R. A. Joladarashi D. Suresh Babu S. Krishnamurthy S. Bhimaraj A. (2016). MicroRNA-9 inhibits hyperglycemia-induced pyroptosis in human ventricular cardiomyocytes by targeting ELAVL1. Biochem. Biophys. Res. Commun. 471, 423–429. 10.1016/j.bbrc.2016.02.065 26898797 PMC4818978

[B28] Joubert M. Manrique A. Cariou B. Prieur X. (2019). Diabetes-related cardiomyopathy: the sweet story of glucose overload from epidemiology to cellular pathways. Diabetes Metab. 45, 238–247. 10.1016/j.diabet.2018.07.003 30078623

[B29] Kam T. I. Mao X. Park H. Chou S. C. Karuppagounder S. S. Umanah G. E. (2018). Poly(ADP-ribose) drives pathologic alpha-synuclein neurodegeneration in Parkinson's disease. Science 362, eaat8407. 10.1126/science.aat8407 30385548 PMC6431793

[B30] Lee C. Do H. T. T. Her J. Kim Y. Seo D. Rhee I. (2019). Inflammasome as a promising therapeutic target for cancer. Life Sci. 231, 116593. 10.1016/j.lfs.2019.116593 31228512

[B31] Li Y. Sun X. Dey S. K. (2015). Entosis allows timely elimination of the luminal epithelial barrier for embryo implantation. Cell Rep. 11, 358–365. 10.1016/j.celrep.2015.03.035 25865893 PMC5089169

[B32] Li X. Wang H. Yao B. Xu W. Chen J. Zhou X. (2016). lncRNA H19/miR-675 axis regulates cardiomyocyte apoptosis by targeting VDAC1 in diabetic cardiomyopathy. Sci. Rep. 6, 36340. 10.1038/srep36340 27796346 PMC5087087

[B33] Li Z. Song Y. Liu L. Hou N. An X. Zhan D. (2017). miR-199a impairs autophagy and induces cardiac hypertrophy through mTOR activation. Cell Death Differ. 24, 1205–1213. 10.1038/cdd.2015.95 26160071 PMC5520159

[B34] Li G. X. Jiao X. H. Cheng X. B. (2018a). Correlations between blood uric acid and the incidence and progression of type 2 diabetes nephropathy. Eur. Rev. Med. Pharmacol. Sci. 22, 506–511. 10.26355/eurrev_201801_14202 29424910

[B35] Li Y. Wang Y. Zou M. Chen C. Chen Y. Xue R. (2018b). AMPK blunts chronic heart failure by inhibiting autophagy. Biosci. Rep. 38 BSR20170982 10.1042/BSR20170982 30021848 PMC6050195

[B36] Li H. Shi Y. Wang X. Li P. Zhang S. Wu T. (2019a). Piceatannol alleviates inflammation and oxidative stress *via* modulation of the Nrf2/HO-1 and NF-kappaB pathways in diabetic cardiomyopathy. Chem. Biol. Interact. 310, 108754. 10.1016/j.cbi.2019.108754 31323227

[B37] Li J. Yuan Y. Q. Zhang L. Zhang H. Zhang S. W. Zhang Y. (2019b). Exogenous hydrogen sulfide protects against high glucose-induced apoptosis and oxidative stress by inhibiting the STAT3/HIF-1alpha pathway in H9c2 cardiomyocytes. Exp. Ther. Med. 18, 3948–3958. 10.3892/etm.2019.8036 31616516 PMC6781810

[B38] Li X. Ke X. Li Z. Li B. (2019c). Vaspin prevents myocardial injury in rats model of diabetic cardiomyopathy by enhancing autophagy and inhibiting inflammation. Biochem. Biophys. Res. Commun. 514, 1–8. 10.1016/j.bbrc.2019.04.110 31014675

[B39] Li Y. Yang Y. Zhao Y. Zhang J. Liu B. Jiao S. (2019d). Astragaloside IV reduces neuronal apoptosis and parthanatos in ischemic injury by preserving mitochondrial hexokinase-II. Free Radic. Biol. Med. 131, 251–263. 10.1016/j.freeradbiomed.2018.11.033 30502455

[B40] Liang W. Chen J. Mo L. Ke X. Zhang W. Zheng D. (2016). ATP-sensitive K(+) channels contribute to the protective effects of exogenous hydrogen sulfide against high glucose-induced injury in H9c2 cardiac cells. Int. J. Mol. Med. 37, 763–772. 10.3892/ijmm.2016.2467 26820501

[B41] Liang W. Chen M. Zheng D. Li J. Song M. Zhang W. (2017). The opening of ATP-Sensitive K+ channels protects H9c2 cardiac cells against the high glucose-induced injury and inflammation by Inhibiting the ROS-TLR4-necroptosis pathway. Cell. Physiol. Biochem. 41, 1020–1034. 10.1159/000461391 28291959

[B42] Lin C. Zhang M. Zhang Y. Yang K. Hu J. Si R. (2017). Helix B surface peptide attenuates diabetic cardiomyopathy *via* AMPK-dependent autophagy. Biochem. Biophys. Res. Commun. 482, 665–671. 10.1016/j.bbrc.2016.11.091 27865838

[B43] Linkermann A. (2016). Nonapoptotic cell death in acute kidney injury and transplantation. Kidney Int. 89, 46–57. 10.1016/j.kint.2015.10.008 26759047

[B44] Liu J. Tang Y. Feng Z. Liu J. Liu J. Long J. (2014). (-)-Epigallocatechin-3-gallate attenuated myocardial mitochondrial dysfunction and autophagy in diabetic Goto-Kakizaki rats. Free Radic. Res. 48, 898–906. 10.3109/10715762.2014.920955 24797301

[B45] Liu Y. Zheng W. Pan Y. Hu J. (2019). Low expression of miR-186-5p regulates cell apoptosis by targeting toll-like receptor 3 in high glucose-induced cardiomyocytes. J. Cell. Biochem. 120, 9532–9538. 10.1002/jcb.28229 30506923

[B46] Martins I. Raza S. Q. Voisin L. Dakhli H. Law F. De Jong D. (2017). Entosis: The emerging face of non-cell-autonomous type IV programmed death. Biomed. J. 40, 133–140. 10.1016/j.bj.2017.05.001 28651734 PMC6136291

[B47] Marunouchi T. Tanonaka K. (2015). Cell death in the cardiac myocyte. Biol. Pharm. Bull. 38, 1094–1097. 10.1248/bpb.b15-00288 26235571

[B48] Meng T. Lin S. Zhuang H. Huang H. He Z. Hu Y. (2019). Recent progress in the role of autophagy in neurological diseases. Cell Stress 3, 141–161. 10.15698/cst2019.05.186 31225510 PMC6551859

[B49] Mitroulis I. Kambas K. Chrysanthopoulou A. Skendros P. Apostolidou E. Kourtzelis I. (2011). Neutrophil extracellular trap formation is associated with IL-1beta and autophagy-related signaling in gout. PloS One 6, e29318. 10.1371/journal.pone.0029318 22195044 PMC3241704

[B50] Nomura M. Ueno A. Saga K. Fukuzawa M. Kaneda Y. (2014). Accumulation of cytosolic calcium induces necroptotic cell death in human neuroblastoma. Cancer Res. 74, 1056–1066. 10.1158/0008-5472.CAN-13-1283 24371227

[B51] Oh J. E. Jun J. H. Hwang H. J. Shin E. J. Oh Y. J. Choi Y. S. (2019). Dexmedetomidine restores autophagy and cardiac dysfunction in rats with streptozotocin-induced diabetes mellitus. Acta Diabetol. 56, 105–114. 10.1007/s00592-018-1225-9 30206697

[B52] Paolillo S. Marsico F. Prastaro M. Renga F. Esposito L. De Martino F. (2019). Diabetic cardiomyopathy: definition, diagnosis, and therapeutic implications. Heart Fail. Clin. 15, 341–347. 10.1016/j.hfc.2019.02.003 31079692

[B53] Parim B. Sathibabu Uddandrao V. V. Saravanan G. (2019). Diabetic cardiomyopathy: molecular mechanisms, detrimental effects of conventional treatment, and beneficial effects of natural therapy. Heart Fail. Rev. 24, 279–299. 10.1007/s10741-018-9749-1 30349977

[B54] Ren X. M. Zuo G. F. Wu W. Luo J. Ye P. Chen S. L. (2016). Atorvastatin alleviates experimental diabetic cardiomyopathy by regulating the GSK-3beta-PP2Ac-NF-kappaB signaling axis. PloS One 11, e0166740. 10.1371/journal.pone.0166740 27851811 PMC5112957

[B55] Robinson N. Ganesan R. Hegedus C. Kovacs K. Kufer T. A. Virag L. (2019). Programmed necrotic cell death of macrophages: Focus on pyroptosis, necroptosis, and parthanatos. Redox Biol. 26, 101239. 10.1016/j.redox.2019.101239 31212216 PMC6582207

[B56] Scaturro P. Pichlmair A. (2018). Oxeiptosis-a cell death pathway to mitigate damage caused by radicals. Cell Death Differ. 25, 1191–1193. 10.1038/s41418-018-0134-3 29844568 PMC6030169

[B57] Song X. Zhu S. Xie Y. Liu J. Sun L. Zeng D. (2018). JTC801 induces pH-dependent death specifically in cancer cells and slows growth of tumors in mice. Gastroenterology 154, 1480–1493. 10.1053/j.gastro.2017.12.004 29248440 PMC5880694

[B58] Sun S. Yang S. Dai M. Jia X. Wang Q. Zhang Z. (2017). The effect of Astragalus polysaccharides on attenuation of diabetic cardiomyopathy through inhibiting the extrinsic and intrinsic apoptotic pathways in high glucose -stimulated H9C2 cells. BMC Complement. Altern. Med. 17, 310. 10.1186/s12906-017-1828-7 28610566 PMC5470251

[B59] Sun L. Chen Y. Luo H. Xu M. Meng G. Zhang W. (2019a). Ca(2+)/calmodulin-dependent protein kinase II regulation by inhibitor 1 of protein phosphatase 1 alleviates necroptosis in high glucose-induced cardiomyocytes injury. Biochem. Pharmacol. 163, 194–205. 10.1016/j.bcp.2019.02.022 30779910

[B60] Sun Q. Wu X. Wang H. Chen W. Zhao X. Yang Y. (2019b). Protective effects of astragalus polysaccharides on oxidative stress in high glucose-induced Or SOD2-Silenced H9C2 cells based On PCR Array Analysis. Diabetes Metab. Syndr. Obes. 12, 2209–2220. 10.2147/DMSO.S228351 31695464 PMC6821059

[B61] Sun S. Yang S. An N. Wang G. Xu Q. Liu J. (2019c). Astragalus polysaccharides inhibits cardiomyocyte apoptosis during diabetic cardiomyopathy *via the* endoplasmic reticulum stress pathway. J. Ethnopharmacol. 238, 111857. 10.1016/j.jep.2019.111857 30959142

[B62] Tan X. Hu L. Shu Z. Chen L. Li X. Du M. (2019). Role of CCR2 in the development of streptozotocin-treated diabetic cardiomyopathy. Diabetes 68, 2063–2073. 10.2337/db18-1231 31439648 PMC6804626

[B63] Tang D. Kang R. Berghe T. V. Vandenabeele P. Kroemer G. (2019a). The molecular machinery of regulated cell death. Cell Res. 29, 347–364. 10.1038/s41422-019-0164-5 30948788 PMC6796845

[B64] Tang S. G. Liu X. Y. Wang S. P. Wang H. H. Jovanovic A. Tan W. (2019b). Trimetazidine prevents diabetic cardiomyopathy by inhibiting Nox2/TRPC3-induced oxidative stress. J. Pharmacol. Sci. 139, 311–318. 10.1016/j.jphs.2019.01.016 30962089

[B65] Wang J. Liu H. Li N. Zhang Q. Zhang H. (2014). The protective effect of fucoidan in rats with streptozotocin-induced diabetic nephropathy. Mar. Drugs 12, 3292–3306. 10.3390/md12063292 24886867 PMC4071577

[B66] Wang F. Gomez-Sintes R. Boya P. (2018). Lysosomal membrane permeabilization and cell death. Traffic 19, 918–931. 10.1111/tra.12613 30125440

[B67] Wang J. Tang Z. Zhang Y. Qiu C. Zhu L. Zhao N. (2019a). Matrine alleviates AGEs- induced cardiac dysfunctions by attenuating calcium overload *via* reducing ryanodine receptor 2 activity. Eur. J. Pharmacol. 842, 118–124. 10.1016/j.ejphar.2018.10.010 30339815

[B68] Wang L. Yuan D. Zheng J. Wu X. Wang J. Liu X. (2019b). Chikusetsu saponin IVa attenuates isoprenaline-induced myocardial fibrosis in mice through activation autophagy mediated by AMPK/mTOR/ULK1 signaling. Phytomedicine 58, 152764. 10.1016/j.phymed.2018.11.024 31005723

[B69] Wang X. Pan J. Liu D. Zhang M. Li X. Tian J. (2019c). Nicorandil alleviates apoptosis in diabetic cardiomyopathy through PI3K/Akt pathway. J. Cell. Mol. Med. 23, 5349–5359. 10.1111/jcmm.14413 31131539 PMC6653072

[B70] Wang X. Pan J. Liu H. Zhang M. Liu D. Lu L. (2019d). AIM2 gene silencing attenuates diabetic cardiomyopathy in type 2 diabetic rat model. Life Sci. 221, 249–258. 10.1016/j.lfs.2019.02.035 30790610

[B71] Wang Y. Luo W. Wang Y. (2019e). PARP-1 and its associated nucleases in DNA damage response. DNA Repair (Amst) 81, 102651. 10.1016/j.dnarep.2019.102651 31302005 PMC6764844

[B72] Wang Z. Li J. Wang Y. Liu Q. (2019f). Palbociclib improves cardiac dysfunction in diabetic cardiomyopathy by regulating Rb phosphorylation. Am. J. Transl. Res. 11, 3481–3489. 31312360 PMC6614619

[B73] Wei H. Qu H. Wang H. Ji B. Ding Y. Liu D. (2017). 1,25-Dihydroxyvitamin-D3 prevents the development of diabetic cardiomyopathy in type 1 diabetic rats by enhancing autophagy *via* inhibiting the beta-catenin/TCF4/GSK-3beta/mTOR pathway. J. Steroid. Biochem. Mol. Biol. 168, 71–90. 10.1016/j.jsbmb.2017.02.007 28216152

[B74] Wu B. Lin J. Luo J. Han D. Fan M. Guo T. (2017). Dihydromyricetin protects against diabetic cardiomyopathy in streptozotocin-induced diabetic mice. Biomed. Res. Int. 2017, 3764370. 10.1155/2017/3764370 28421194 PMC5379084

[B75] Wu A. Hu P. Lin J. Xia W. Zhang R. (2018a). Activating Cannabinoid receptor 2 protects against diabetic cardiomyopathy through autophagy induction. Front. Pharmacol. 9, 1292. 10.3389/fphar.2018.01292 30459625 PMC6232417

[B76] Wu M. Y. Yiang G. T. Liao W. T. Tsai A. P. Cheng Y. L. Cheng P. W. (2018b). Current mechanistic concepts in ischemia and reperfusion injury. Cell Physiol. Biochem. 46, 1650–1667. 10.1159/000489241 29694958

[B77] Xiao F. J. Zhang D. Wu Y. Jia Q. H. Zhang L. Li Y. X. (2019). miRNA-17-92 protects endothelial cells from erastin-induced ferroptosis through targeting the A20-ACSL4 axis. Biochem. Biophys. Res. Commun. 515, 448–454. 10.1016/j.bbrc.2019.05.147 31160087

[B78] Xing R. Liu D. Cheng X. Tian X. Yan C. Han Y. (2019). MiR-207 inhibits autophagy and promotes apoptosis of cardiomyocytes by directly targeting LAMP2 in type 2 diabetic cardiomyopathy. Biochem. Biophys. Res. Commun. 520, 27–34. 10.1016/j.bbrc.2019.09.092 31564413

[B79] Xu X. Kobayashi S. Chen K. Timm D. Volden P. Huang Y. (2013). Diminished autophagy limits cardiac injury in mouse models of type 1 diabetes. J. Biol. Chem. 288, 18077–18092. 10.1074/jbc.M113.474650 23658055 PMC3689952

[B80] Xu K. Liu X. F. Ke Z. Q. Yao Q. Guo S. Liu C. (2018). Resveratrol modulates apoptosis and autophagy induced by high glucose and palmitate in cardiac cells. Cell Physiol. Biochem. 46, 2031–2040. 10.1159/000489442 29723857

[B81] Xue M. Li T. Wang Y. Chang Y. Cheng Y. Lu Y. (2019). Empagliflozin prevents cardiomyopathy *via* sGC-cGMP-PKG pathway in type 2 diabetes mice. Clin. Sci. (Lond) 133, 1705–1720. 10.1042/CS20190585 31337673

[B82] Yang Y. P. Liang Z. Q. Gu Z. L. Qin Z. H. (2005). Molecular mechanism and regulation of autophagy. Acta Pharmacol. Sin. 26, 1421–1434. 10.1111/j.1745-7254.2005.00235.x 16297339

[B83] Yang W. S. SriRamaratnam R. Welsch M. E. Shimada K. Skouta R. Viswanathan V. S. (2014). Regulation of ferroptotic cancer cell death by GPX4. Cell 156, 317–331. 10.1016/j.cell.2013.12.010 24439385 PMC4076414

[B84] Yang F. Qin Y. Wang Y. Meng S. Xian H. Che H. (2019). Metformin inhibits the NLRP3 Inflammasome *via* AMPK/mTOR-dependent effects in diabetic cardiomyopathy. Int. J. Biol. Sci. 15, 1010–1019. 10.7150/ijbs.29680 31182921 PMC6535781

[B85] Yao Q. Ke Z. Q. Guo S. Yang X. S. Zhang F. X. Liu X. F. (2018). Curcumin protects against diabetic cardiomyopathy by promoting autophagy and alleviating apoptosis. J. Mol. Cell. Cardiol. 124, 26–34. 10.1016/j.yjmcc.2018.10.004 30292723

[B86] Yu W. Gao B. Li N. Wang J. Qiu C. Zhang G. (2017). Sirt3 deficiency exacerbates diabetic cardiac dysfunction: role of Foxo3A-Parkin-mediated mitophagy. Biochim. Biophys. Acta Mol. Basis. Dis. 1863, 1973–1983. 10.1016/j.bbadis.2016.10.021 27794418

[B87] Zeng C. Wang R. Tan H. (2019). Role of pyroptosis in cardiovascular diseases and its therapeutic implications. Int. J. Biol. Sci. 15, 1345–1357. 10.7150/ijbs.33568 31337966 PMC6643148

[B88] Zhang T. Zhang Y. Cui M. Jin L. Wang Y. Lv F. (2016). CaMKII is a RIP3 substrate mediating ischemia- and oxidative stress-induced myocardial necroptosis. Nat. Med. 22, 175–182. 10.1038/nm.4017 26726877

[B89] Zhang B. Zhang J. Zhang C. Zhang X. Ye J. Kuang S. (2018). Notoginsenoside R1 protects against diabetic cardiomyopathy through activating estrogen receptor alpha and its downstream signaling. Front. Pharmacol. 9, 1227. 10.3389/fphar.2018.01227 30450046 PMC6224485

[B90] Zhao Z. Guo L. Shi W. Zuo W. (2019). Role of pyroptosis in cardiovascular disease. Cell Prolif. 52, e12563. 10.1111/cpr.12563 30525268 PMC6496801

[B91] Zhou H. Li D. Zhu P. Ma Q. Toan S. Wang J. (2018a). Inhibitory effect of melatonin on necroptosis *via* repressing the Ripk3-PGAM5-CypD-mPTP pathway attenuates cardiac microvascular ischemia-reperfusion injury. J. Pineal. Res. 65, e12503. 10.1111/jpi.12503 29770487

[B92] Zhou Y. Wang H. Man F. Guo Z. Xu J. Yan W. (2018b). Sitagliptin protects cardiac function by reducing nitroxidative stress and promoting autophagy in Zucker Diabetic Fatty (ZDF) rats. Cardiovasc. Drugs Ther. 32, 541–552. 10.1007/s10557-018-6831-9 30328028

[B93] Zou F. Wang L. Liu H. Wang W. Hu L. Xiong X. (2019a). Sophocarpine suppresses NF-kappaB-mediated inflammation both in vitro and in vivo and inhibits diabetic cardiomyopathy. Front. Pharmacol. 10, 1219. 10.3389/fphar.2019.01219 31736745 PMC6836764

[B94] Zou G. Zhong W. Wu F. Wang X. Liu L. (2019b). Catalpol attenuates cardiomyocyte apoptosis in diabetic cardiomyopathy *via* Neat1/miR-140-5p/HDAC4 axis. Biochimie 165, 90–99. 10.1016/j.biochi.2019.05.005 31078585

